# The number of intracorneal ring segments in asymmetric and central cones

**DOI:** 10.1186/s40662-021-00234-6

**Published:** 2021-03-30

**Authors:** Canan Asli Utine, Denizcan Özizmirliler, Mustafa Kayabaşı, Üzeyir Günenç

**Affiliations:** 1 Department of Ophthalmology, Faculty of Medicine, Dokuz Eylul University, Mithatpasa cad, No:1606 Inciraltı Kampusu, Balcova, 35330 Izmir, Turkey; 2 Izmir Biomedicine and Genome Center, Izmir, Turkey

**Keywords:** Cone eccentricity, Intracorneal ring segment implantation, Keratoconus, Nomogram

## Abstract

**Background:**

To compare the results of single versus double intracorneal ring segment (ICRS) (KeraRing) implantation in keratoconus with respect to different cone locations.

**Methods:**

Twenty-two eyes of 18 patients with totally asymmetric cones (20–80% or 0–100% distribution along steep axis) were implanted with single ICRS (Group 1), 38 eyes of 32 patients with central or partially asymmetric cones (50–50% or 40–60% distribution along steep axis) were implanted with double ICRS (Group 2), at a depth of 80% of the site of implantation, in channels created with femtosecond laser device. All patients had uncorrected and corrected distance visual acuities (UDVA and CDVA, respectively) of ≤ 0.3 Snellen lines.

**Results:**

In both groups, patients had median UDVA and CDVA gain of 3 Snellen lines (*P* > 0.05). Postoperative improvement in indices of vertical asymmetry and height decentration in Group 1; simulated keratometry, corneal astigmatism and anterior corneal asphericity values in Group 2 were greater (*P* < 0.05). A total of 10 eyes (45.5%) in Group 1 were recommended double ring implantation by the manufacturer’s nomogram, but underwent single ICRS implantation and achieved visual, refractive, tomographic outcomes comparable to that in Group 2, although corneal cylindrical correction was less and final topographic astigmatism was greater.

**Conclusion:**

Double ICRS implantation seems to be superior in terms of keratometry, corneal astigmatism and anterior corneal asphericity improvement. Single ICRS implantation in totally asymmetric cones seems to provide satisfactory visual, refractive and tomographic results, similar to double ICRS implantation in central and partially asymmetric cones, by inducing central shift of the cone.

## Introduction

Intracorneal ring segments (ICRS) have long been used for the optical rehabilitation of ectatic corneal disorders alone or in combination with other surgical procedures [1, 2]. The ultimate aim is to provide regularization of the corneal surface [3] and improve its asphericity [4], and a decrease in refractive error, for subsequent visual improvement.

Nomograms are clinical guidelines to determine the number of ring segments to implant, their arc lengths and thicknesses as well as location of insertion, based on preoperative parameters [5]. However, nomograms are not accurate in all cone types, as a theoretical nomogram cannot address all possibilities. Most nomograms are empirical and do not correspond to an accurate mathematical model of ICRS effect on the ectatic cornea [6]. As nomograms are far from predictability in their current states, postoperative unwanted refractive and visual surprises are common. Currently, a lot of effort is put into improving nomograms to better reflect biomechanical behavior of ectatic cornea to ICRS implantation and to improve predictability of final refractive and visual outcomes [7, 8]. Clinical feedback is important in this sense.

Herein, we report our results of eyes with keratoconus that were implanted with single or double ring segments depending on cone location. The purpose of this study is to explore whether single ICRS implantation can yield similar efficacy in eyes with totally asymmetric cones, as compared to double ICRS implantation in eyes with central or partially asymmetric cones.

## Materials and methods

This study includes retrospective evaluation of the cohort implanted with ICRS at Dokuz Eylül University, Department of Ophthalmology, Cornea Division, between February 2016 to February 2020. The study adhered to the Tenets of the Declaration of Helsinki. Dokuz Eylül University Ethical Committee Approval was obtained for this research (2019/23–42).

Patients with keratoconus who were admitted for optical and visual rehabilitation underwent a complete ophthalmic examination that included assessment of uncorrected and corrected distance visual acuities (UDVA and CDVA, respectively) with spherical and cylindrical refraction, and Scheimpflug tomography including pachymetry map (Pentacam, Oculus®, Germany). For patients who wore contact lenses, discontinuation was advised prior to the examination for at least 2 weeks for soft contact lenses and a month for rigid contact lenses.

Given the unpredictable nature of visual results of ICRS surgery, we advocated ICRS implantation only in eyes with preoperative CDVA of ≤ 0.3 Snellen lines by spectacle correction or soft/ rigid gas permeable keratoconus contact lens fit, but SimK_avg_ of ≤ 60 D. Patients considered for ICRS implantation surgery were at least 18 years old, had central clear corneas, corneal pachymetry ≥ 400 μm at the site of the corneal channel (depending on the thickness of ICRS to be implanted), scotopic pupil diameter < 5 mm, and displayed evidence of aligned refractive and keratometric axes (i.e., the flattest corneal meridian and the refractive cylinder axis expressed as a negative value formed an angle between 0° and 15°). Contraindications included existing collagen vascular, autoimmune, or immunodeficiency diseases, atopy, diabetes or pregnancy within the past 1 year, and ocular co-morbidities including altered eyelid anatomy and function, ocular surface diseases, co-existing corneal dystrophies, ocular media opacities, glaucoma, or vitreoretinal disorders. After a thorough discussion on possible risks and benefits, ICRS implantation was planned. Detailed signed informed consent was obtained from each subject before the surgery.

### Surgical technique

KeraRing (Mediphacos, Brazil) segments are ring segments made of polymethyl-methacrylate with a triangular transverse section, optical zone of 5.0 mm, inner and outer diameters of 5.40 mm and 6.60 mm, respectively, base width of 800 μm, thicknesses of 150–300 μm with 50 μm increments and arc lengths of 90°–210°. Selection of ICRS parameters was made as per the KeraRing 2009 nomogram [9] based on visual, refractive and topographic parameters. The number of ring segments were determined according to the distribution of ectatic area on the corneal surface, whereas the thickness and arc lengths of the ring segments were determined according to the spherocylindrical refraction and CDVA. The intrastromal channels for ICRS implantation were created with a femtosecond laser device (iFS, Advanced Femtosecond laser, Abbott Laboratories Inc., Abbott Park, Illinois, USA) with ring energy and side cut energy of 1.30 μJ, inner and outer diameters of 5.0 mm and 5.7 mm, respectively, and at 80% depth of the thinnest stromal thickness at the implantation site. Corneal pachymetry safety limits over implanted ring segments were considered as per the nomogram, to determine the thickness of ring segments to be implanted. Selection of the ring parameters, and ICRS implantation were performed by the same surgeon (CAU).

Location of the cone was defined as described in the nomogram [9]. As CDVA was ≤ 0.3 in all cases, treatment was planned on the keratometric values and steep axis. A reference line was drawn along the steep meridian on the sagittal topography map. Corneal asymmetry type was determined by studying the steep area on each side of the reference meridian [9]. If the line separated the steep area into two equal parts, cone location was said to be “central”. If the line divided the steep area into 40% and 60% segments, cone location was said to be “partially asymmetric”. If the line divided the steep area into 20% and 80% segments or if the steep area retained 100% at one side of the line, cone location was said to be “totally asymmetric”. Corneas with “totally asymmetric” cone location were implanted with a single ring segment regardless of the spherical refractive error and nomogram recommendations. Patients were assured that based on postoperative refraction, visual acuity and topography results, the 2nd ring segment could be implanted in the superior half of the created circular intracorneal channel in the following 3 months. Corneas with “central” or “partially asymmetric” cones were implanted with double rings as indicated by the nomogram.

The postoperative regimen included moxifloxacin 0.5% (Vigamox®, Alcon, USA) and dexamethasone 0.1% (Maxidex®, Alcon, USA) eye drops four times daily for 1 week. The antibiotic eyedrop was discontinued after 1 week, and steroid eyedrop was gradually tapered to be discontinued in 1 month in all eyes. Patients were instructed to avoid eye rubbing and to use preservative-free sodium hyaluronate 15% (Eyestil®; SIFI, Italy), as needed.

This study included patients whose keratoconus were not progressive, and thus additional cross-linking surgery was not performed. Additionally, patients who had cross-linking surgery before were not included into this study, as the biomechanical behavior of the cornea in response to ICRS implantation surgery might be affected by previous cross-linking. Eyes with any complications requiring ring revision or explantation were not included into the study.

Complete ophthalmologic examination was repeated postoperatively at the 3rd month, when final refractive and visual results are expected to be achieved. Changes in UDVA and CDVA in Snellen lines; spherical, cylindrical and spherical equivalent (SE) of refractive errors; mean, steepest and flattest simulated keratometry readings (SimK_avg_, SimK_s_, SimK_f_, respectively), as well as anterior and posterior asphericity values (Q_ant_ and Q_post_, respectively) and topometric indices measured with Scheimpflug tomography were evaluated. The relationship between these parameters and the number of implanted ICRS were analyzed retrospectively.

### Statistical analysis

The statistical analysis of the data was performed by one of the authors (CAU) using Microsoft Excel 2016. The values were expressed as mean ± standard deviation and a paired sample Student’s t-test was used to analyze changes induced by the surgery, in each group. An independent sample Student’s t-test was used to compare preoperative and postoperative data, as well as surgically induced changes in separate two groups. *P* values < 0.05 were considered statistically significant.

Vectorial analysis of induced cylindrical correction at the cornea was performed with Alpins’ method [10], by using the software (Astigmatizma Analizinde Vektöryel Analiz Programı v.2.0) developed for this purpose at the Department of Ophthalmology, Ege University, Turkey [11].

Safety and efficacy indices were defined as follows, and were calculated for each group:

$$ Safety\ Index=\frac{Mean\ postoperative\ CDVA}{Mean\ preoperative\ CDVA} $$

$$ Efficacy\ index=\frac{Mean\ postoperative\ UDVA}{Mean\ preoperative\ CDVA} $$

A subgroup analysis was performed to evaluate the eyes that achieved satisfactory clinical improvement with a single ring segment, in spite of the two ring segments recommended by the manufacturer’s nomogram. This subgroup was also compared with Group 2, separately.

## Results

A total of 22 eyes of 18 patients with totally asymmetric cones were implanted with a single ICRS and formed Group 1; whereas 38 eyes of 32 patients with central or partially asymmetric cones were implanted with double ICRS and formed Group 2. Baseline characteristics of the patients in Group 1 and Group 2 are shown in Table 1. Preoperatively, mean SimK_avg_ and SimK_s_ were greater in Group 2 (*P* < 0.05), whereas mean index of vertical asymmetry (IVA) was greater in Group 1 (*P* = 0.01).

**Table 1:** Visual, refractive and tomographic parameters of patients who were implanted with single ICRS (Group 1) and double ICRS (Group 2)

	Preoperative			Postoperative			Changes induced by the surgery		
	Group 1	Group 2	P value	Group 1	Group 2	P value	Group 1	Group 2	P value
**UDVA (Snellen lines)**	0.11 ± 0.10	0.09 ± 0.09	0.67	0.41 ± 0.24	0.35 ± 0.23	0.34	0.31 ± 0.22	0.26 ± 0.25	0.45
**CDVA (Snellen lines)**	0.35 ± 0.20	0.29 ± 0.12	0.13	0.64 ± 0.25	0.56 ± 0.20	0.14	0.29 ± 0.21	0.27 ± 0.20	0.69
**Spherical refraction (D)**	−2.48 ± 3.73	−4.19 ± 4.49	0.16	−1.76 ± 3.41	−2.69 ± 2.97	0.28	0.49 ± 2.70	1.39 ± 4.25	0.38
**Cylindrical refraction (D)**	−4.98 ± 2.13	−5.68 ± 2.67	0.32	−2.12 ± 1.54	−2.39 ± 1.67	0.56	2.50 ± 2.17	3.52 ± 2.97	0.17
**SE of refractive error (D)**	− 4.73 ± 4.31	−7.03 ± 4.06	0.05	−2.77 ± 3.64	−3.70 ± 3.18	0.32	1.74 ± 2.71	3.15 ± 4.31	0.18
**SimK**_**avg**_ **(D)**	49.10 ± 3.67	51.37 ± 3.68	0.03*	47.13 ± 3.90	48.29 ± 3.08	0.21	−1.98 ± 1.90	−3.11 ± 2.05	0.04*
**SimK**_**f**_ **(D)**	47.17 ± 3.93	48.78 ± 3.95	0.14	45.76 ± 3.72	47.19 ± 3.12	0.12	−1.40 ± 1.56	−1.51 ± 2.39	0.85
**SimK**_**s**_ **(D)**	51.67 ± 4.41	54.30 ± 3.61	0.02*	48.61 ± 4.15	49.47 ± 3.19	0.38	−3.06 ± − 1.56	−4.83 ± 2.19	< 0.01*
**Central pachymetry (μm)**	461.73 ± 41.65	444.92 ± 47.91	0.18	479.0 ± 48.39	457.11 ± 50.09	0.11	17.27 ± 13.86	12.18 ± 18.75	0.28
**Thinnest pachymetry (μm)**	440.68 ± 44.27	432.82 ± 49.69	0.55	463.59 ± 46.53	434.82 ± 55.01	0.05	22.91 ± 17.39	2.00 ± 35.23	0.01*
**Q**_**ant**_	−0.91 ± 0.47	−1.12 ± 0.39	0.07	−0.61 ± 0.51	−0.57 ± 0.52	0.79	0.30 ± 0.22	0.56 ± 0.44	0.01*
**Q**_**post**_	−0.92 ± 0.46	−1.11 ± 0.56	0.19	−0.86 ± 0.43	−1.13 ± 0.56	0.07	0.06 ± 0.15	0.00 ± 0.50	0.64
**ISV**	95.41 ± 35.20	91.39 ± 28.93	0.64	75.18 ± 30.19	82.19 ± 29.87	0.40	−20.23 ± 13.67	−11.37 ± 21.53	0.09
**IVA**	0.99 ± 0.43	0.70 ± 0.39	0.01*	0.73 ± 0.41	0.72 ± 0.40	0.88	−0.25 ± 0.20	−0.01 ± 0.26	< 0.01*
**KI**	1.26 ± 0.12	1.20 ± 0.12	0.09	1.18 ± 0.12	1.13 ± 0.13	0.16	−0.08 ± 0.05	−0.10 ± 0.20	0.59
**CKI**	1.07 ± 0.05	1.09 ± 0.05	0.18	1.06 ± 0.05	1.10 ± 0.06	0.01*	−0.01 ± 0.03	0.02 ± 0.19	0.93
**IHA**	19.06 ± 23.40	26.76 ± 20.96	0.20	22.22 ± 15.37	27.14 ± 19.50	0.32	3.16 ± 23.87	0.33 ± 23.84	0.59
**IHD**	0.14 ± 0.07	0.10 ± 0.07	0.05	0.10 ± 0.06	0.09 ± 0.05	0.78	−0.04 ± 0.03	−0.01 ± 0.04	0.01*
**Rmin (mm)**	5.98 ± 0.71	5.74 ± 0.61	0.18	6.22 ± 0.76	5.96 ± 0.56	0.14	0.24 ± 0.29	0.06 ± 0.89	0.36
**TKC**	2.68 ± 0.83	2.64 ± 0.58	0.85	2.44 ± 0.54	2.26 ± 0.55	0.28	−0.55 ± 0.50	−0.45 ± 0.79	0.61
**ACD (mm)**	3.61 ± 0.42	3.43 ± 0.39	0.10	3.46 ± 0.50	3.30 ± 0.39	0.18	−0.15 ± 0.25	−0.22 ± 0.57	0.60
**ACV (mm**^**3)**^	190.30 ± 47.84	177.05 ± 26.24	0.18	178.0 ± 31.30	174.86 ± 24.62	0.71	−52.76 ± 86.87	−43.61 ± 75.52	0.68

* *p* < 0.05 indicating statistical significance

*UDVA=* uncorrected distance visual acuity

*CDVA=* corrected distance visual acuity

*SE=* spherical equivalent

*SimK*_*avg*_ = average simulated keratometry

*SimK*_*f*_ = flat simulated keratometry

*SimK*_*s*_ = steep simulated keratometry

*Q*_*ant*_ = anterior corneal asphericity

*Q*_*post*_ = posterior corneal asphericity

*ISV=* index of surface variance

*IVA=* index of vertical asymmetry

*KI=* keratoconus index

*CKI=* central keratoconus index

*IHA=* index of height asymmetry

*IHD=* index of height decentration

*R*_*min*_ = radius of minimum axial/sagittal curvature

*TKC=* topographic keratoconus classification

*ACD=* anterior chamber depth

*ACV=* anterior chamber volume

**Fig. 1:**
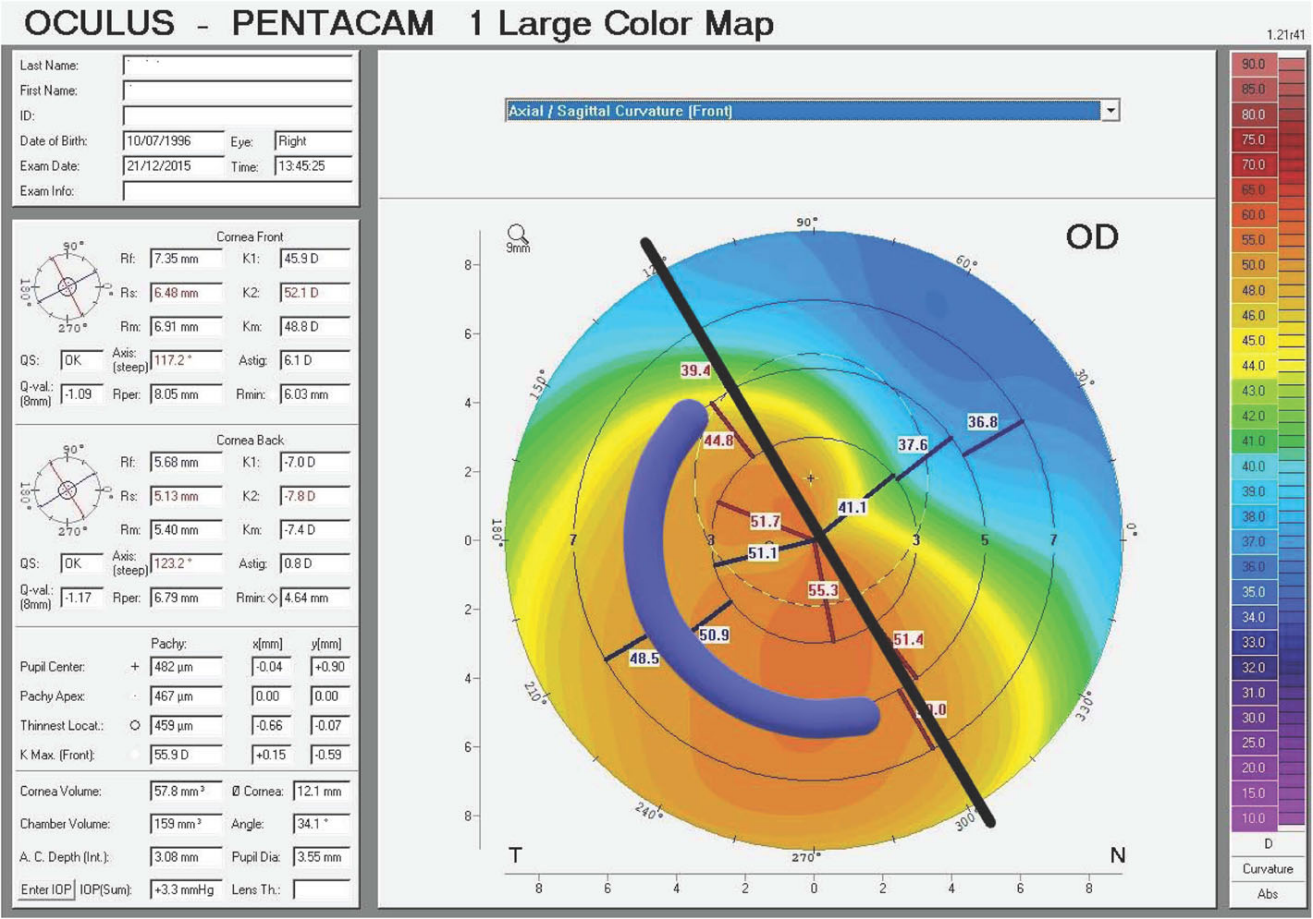
Illustration of an example of totally asymmetric cone and the location where the ring segment was implanted with respect to cone location

Thicknesses and arc lengths of the ICRS implanted in this study ranged between 150 μm and 300 μm and 160°–210°, respectively, in Group 1; 150–350 μm and 90°–160°, respectively, in Group 2. The location where the ring segments were implanted with respect to cone location is depicted in an example of totally asymmetric cone sagittal topography (Fig. 1).

**Fig. 2:**
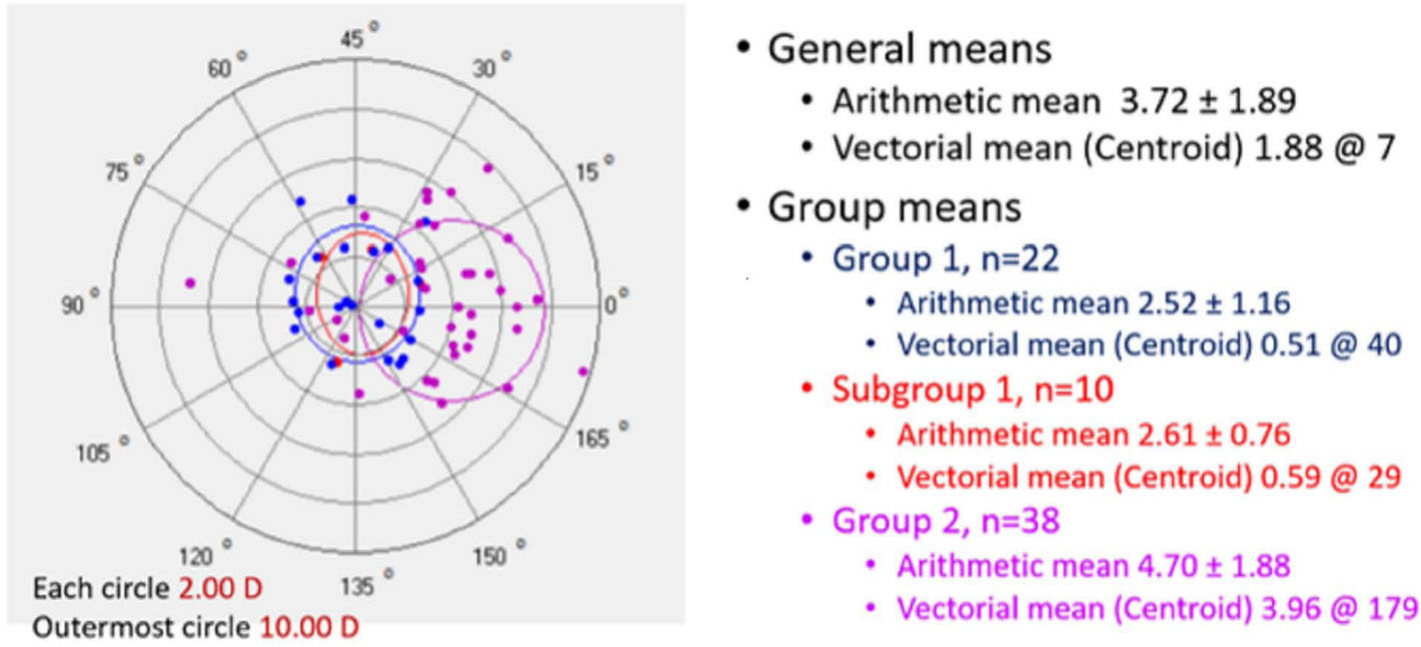
Vectorial analysis of astigmatic correction at the cornea by using keratometric data and Alpins’ method, in Group 1, Group 2 and subgroup of Group 1

**Fig. 3:**
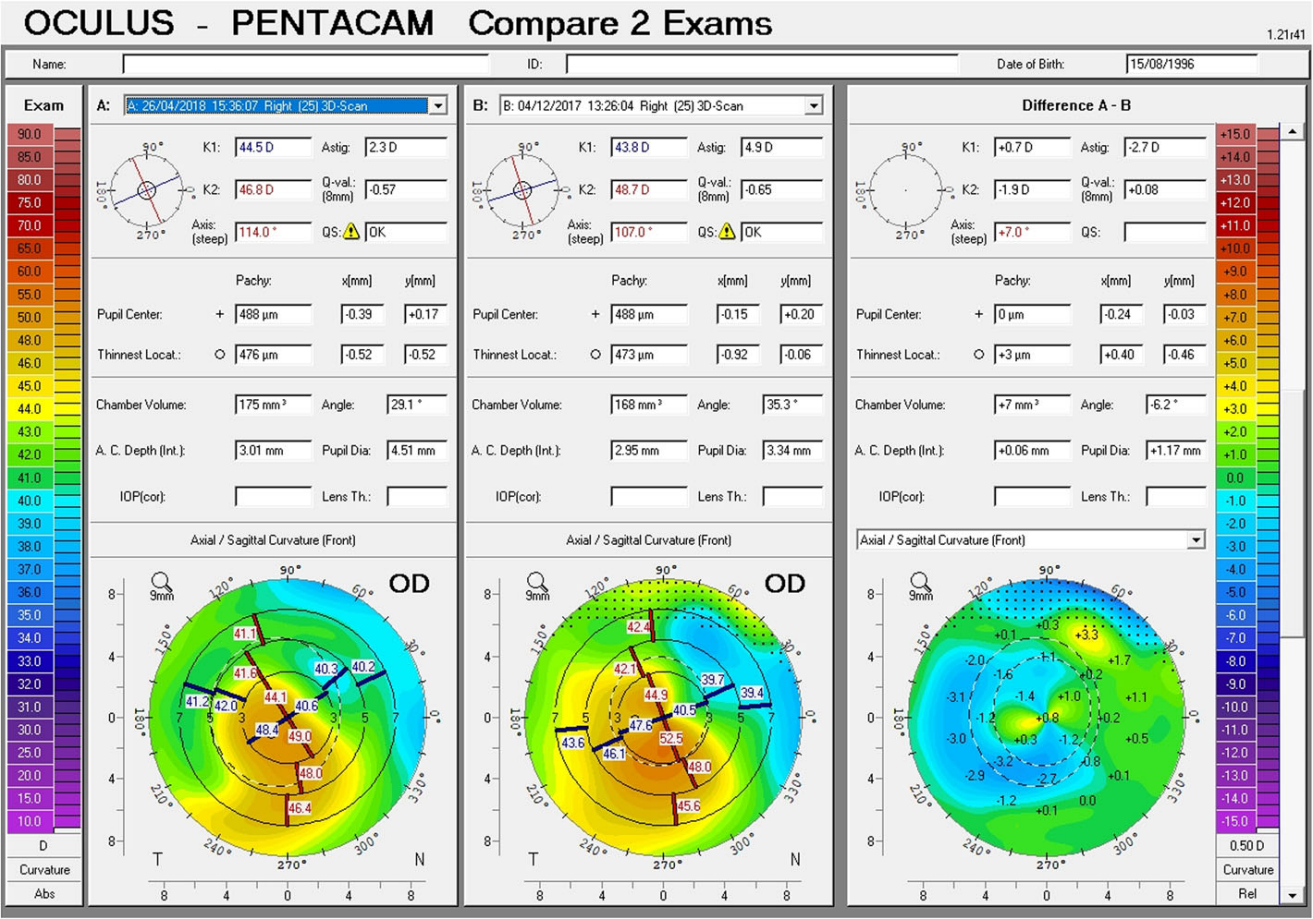
Change in corneal tomography pattern with single ring implantation in a patient with asymmetric cone although the nomogram recommended double ring implantation

**Fig. 4:**
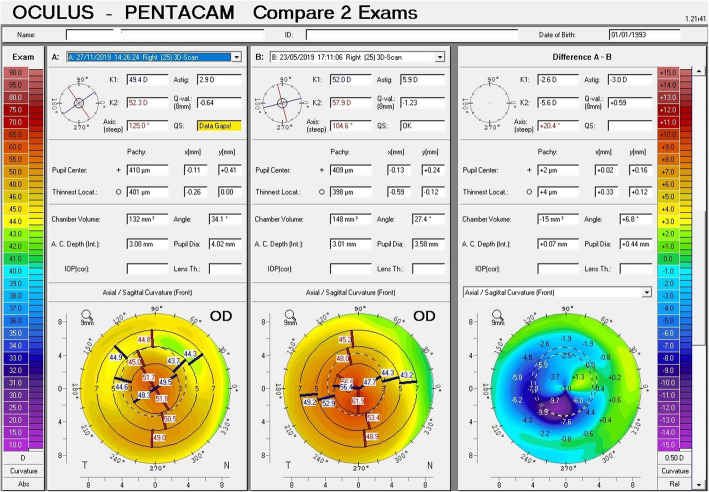
Change in corneal tomography pattern with double ring implantation in a patient with central cone

Postoperative visual, refractive and tomographic parameters of the two groups and changes induced by the surgery are also displayed in Table 1. Importantly, decreases in IVA and index of height decentration (IHD) were greater in Group 1 (*P* ≤ 0.01), indicating improved vertical symmetry of the cornea. Although center – keratoconus index (CKI) was still higher in Group 2 (*P* = 0.01) postoperatively, the decreases in SimK_avg_ and SimK_s_ were greater in Group 2 (*P* < 0.05), indicating greater reduction of the overall curvature. Vectorial analysis of the keratometric changes revealed that astigmatic correction at the cornea was also greatest in Group 2 (Fig. 2). Furthermore, change in anterior corneal asphericity (Q_ant_) was greater in Group 2 (P = 0.01), indicating greater normalization of the corneal asphericity index. Notably, the improvement in Q_ant_ was greater than the improvement in posterior corneal asphericity (Q_post_), in both groups. Safety index of the procedure was 1.83 in Group 1 and 1.93 in Group 2. Efficacy index was 1.17 in Group 1 and 1.21 in Group 2. Despite more favorable tomographic outcomes in Group 2, the postoperative visual and refractive outcomes were similar in both groups (*P* > 0.05, Table 1). Examples of comparative corneal tomographies after single or double ICRS implantation in Group 1 and 2, respectively are shown in Figs. 3 and 4.

A total of 10 eyes (45.5%) in Group 1 were recommended for double ring implantation by the manufacturer’s nomogram, but achieved satisfactory visual, refractory and tomographic improvement with a single ICRS implantation (Fig. 3). These eyes all had asymmetric cones with preoperative SimK_avg_ and topographic astigmatism of 50.85 ± 2.68 D and 5.16 ± 1.06 D, respectively. They improved postoperatively to 48.65 ± 2.50 D and 3.24 ± 0.99 D, respectively (*P* < 0.001). In this subgroup of patients, the preoperative UDVA, CDVA and SE of refraction of 0.08 ± 0.07 D, 0.31 ± 0.14 D and −6.68 ± 2.90 D, respectively; and improved postoperatively to 0.33 ± 0.21 D, 0.56 ± 0.23 D and −4.60 ± 3.94 D, respectively (*P* < 0.01 for all). No eyes in Group 2 had been initially implanted with a single ring and then necessitated implantation of the 2nd ring.

The data of subgroup of 10 eyes that were implanted with a single ring segment although the nomogram recommended two rings was also compared with Group 2, in which the eyes were implanted with two ring segments as the nomogram suggested. Subgroup versus Group 2 analysis revealed that preoperative IVA (1.10 ± 0.32 and 0.70 ± 0.39, respectively), keratoconus index (KI) (1.29 ± 0.10 and 1.20 ± 0.12, respectively) and IHD (0.15 ± 0.05 and 0.10 ± 0.07, respectively) were significantly greater in the subgroup (*P* < 0.05). The amount of cylindrical correction at the cornea in this subgroup was similar to that of Group 1, and less than Group 2 (Fig. 2). However, all postoperative visual, refractive, tomographic parameters and indices were statistically similar (*P* > 0.05), except for topographic astigmatism of 3.24 ± 0.99 D in subgroup and 2.28 ± 1.45 D in Group 2 (*P* = 0.03).

None of the cases in either groups reported visual dissatisfaction or required further corneal interventions including corneal transplantation surgery.

## Discussion

The results of this study revealed that single ICRS implantation in corneas with totally asymmetric cone location yielded improved normalization of vertical asymmetry and height decentration indices, due to a central shift of the cone. Despite more favorable tomographic outcomes including keratometry, corneal astigmatism and anterior corneal asphericity in eyes with central or partially asymmetric cones and double ICRS implantation; postoperative visual and refractive outcomes were similar in both groups.

Factors directly associated with visual improvement after ICRS surgery are not fully elucidated. Remodeling of anterior and posterior corneal topographies improves optical quality of the cornea, reduces optical aberrations and improves CDVA [12]. Recent evidence suggests that normalization of the corneal asphericity [4] has a role in visual improvement. Central shift of the decentered cone and relocation of the relatively more prolate cone area towards the visual axis [13] might also be one of the reasons of improved visual quality.

Nomograms for ICRS selection are proposed by the rings’ manufacturers to guide in choosing the appropriate segment to induce desirable flattening effect and correction of corneal astigmatism and subsequent visual improvement [14]. Although the surgical aspect of ICRS implantation is well mastered, nomograms still cannot predict the exact flattening power of the ring implanted in an ectatic cornea. They are based largely on spherocylindrical refraction and topographic keratometric data, as well as cone location, all of which are objective but may demonstrate fluctuations in keratoconic eyes [15]. Reliable and repeatable data are essential for postoperative predictability, as data entered into the nomograms provide recommendations for the size, thickness, number of the ring segments, spatial location for implantation including stromal depth and vertical incision. In general, the steepest keratometric axis or axis of coma aberration are considered to be the most suitable site to place the vertical incision [16–18]. Furthermore, ICRS inserted deep (i.e., ~ 80%) in posterior stroma was found to be the most effective parameter to alter corneal optics, as rings that act as restraint elements relax stromal stress in cone area [7]. However, controversies still exist on the number of rings to use [19–21].

Some authors initially proposed using two segments of 120° or 160° arc length [22, 23] with equal thicknesses that vary based on the targeted correction of myopic SE [15], while manufacturers initially proposed implantation of two segments with equal arc lengths, with different thickness and distribution of each segment based on SE and topographic pattern [13]. As equal ring size had more flattening effect in the steep area than in the flat area, thicker segment was always implanted at the steep area regardless of the cylinder axis of refraction [24]. Similarly, nomograms tend to place the second ring segment based on targeted SE correction and differ the thicknesses of two segments based on eccentricity and astigmatism to be corrected.

Cone location has a direct role in surgical planning and resultant visual acuity improvement, and is mostly evaluated by topography maps. The cone apex is known to reside at the end of maximum steepness in sagittal topography maps, with immediate re-steepening on the other side of apex. Indeed, cone location is best determined by elevation maps. We propose that this fact is the basis of observed adequacy by implanting only one ring segment in eccentric cones where the reference line divides the steep area into 20% and 80% segments on the sagittal map but the cone apex lies 100% at one side of the reference axis (Fig. 1). As such, no impact of the cone eccentricity on visual outcomes has been detected after KeraRing implantation [25]. In our cohort, we obtained satisfactory visual gain in 10 eyes (45.5%) with a single ring segment implantation, despite nomogram-recommended two ring segments. This deviation from the nomogram was attempted as authors expected clinical improvement with only one segment implantation due to asymmetric cone location, regardless of preoperative SE.

It has been accepted that two symmetrical ring segments create maximum flattening of the central cone [6, 15, 19], whereas asymmetry of segments create astigmatic correction and is preferred in oval cones. In inferior cones with steepening and superior flattening, double ring segments flatten the cornea inferiorly, as well as superiorly. Therefore, a single ring may be a better option based on the topographic profile, to induce inferior flattening and superior steepening resulting in greater change in inferior / superior ratio, and thus greater regularization of corneal surface [6, 15, 18, 21]. In this aspect, Alió et al. was first to report their findings on the implantation of one or two Intacs segments, where they concluded that based on the topographic pattern, best choice was to implant one segment in those cases of inferior steepening and two segments in central cones [19]. Sharma et al. also reported favorable effects of single segment Intacs implantation in keratoconus [21], particularly in moderate and severe asymmetric keratoconus defined as a difference between the superior and inferior topographic readings of > 5 D and an apex topographic reading of ≥ 52 D [24].

Herein, our approach was to first implant a single ring segment in all eyes with “totally asymmetric cones” regardless of the preoperative SE and then decide on the necessity of 2nd ring implantation with the help of postoperative results. Indeed, there were no eyes in Group 2 that were initially implanted with a single ring and then necessitated implantation of the 2nd ring. The study of Alió et al. [19] used the manual ring implantation technique, while we used femtosecond laser to create intrastromal channels at homogenous depth. Our ring parameters varied depending on steepness of the cornea and magnitude of the refractive errors, and were not standard in all cases. Furthermore, this is the first study that compared one or two ring segment implantation with smaller diameter and triangular cross-section (i.e., the KeraRing).

In our cohort, the mean spherical refractive error decreased by 0.49 ± 2.70 D in Group 1, as compared to 1.39 ± 4.25 D in Group 2 (*P* > 0.05). Similarly, the decrease in cylindrical refraction was 2.50 ± 2.17 D in Group 1, and 3.52 ± 2.97 D in Group 2 (P > 0.05). Vectoral analysis of the corneal cylindrical correction yielded 0.51 D at 40° in Group 1 and 3.96 D at 179° in Group 2 (Fig. 2). Yet, in both groups, both UDVA and CDVA increased by a median of 3 Snellen lines. It seems that for visual improvement, spherical or cylindrical refractive error need not be decreased till emmetropia. In this improvement, role of not only the decrease in keratometric readings and subsequent decrease in refractive error, but also the relocation of cone apex towards visual axis, optimized corneal asphericity (i.e., Q values) as well as topometric indices including CKI, IVA, IHD should be explored. We have previously shown that ICRS implantation seems to approximate the anterior corneal asphericity of “advanced prolate” shape to “the optimal prolate” Q value of −0.52 and “spherical aberration-free” human corneal Q value of −0.46 [4]. We also suggested that the preoperative index of surface variance (ISV) value seems to be beneficial in predicting visual gain after ring surgery in addition to SimK_avg_ [9]. In this study, Group 2 had initially higher CKI values, indicating a more severe stage of keratoconus; and this difference persisted postoperatively (Table 1). Among the indices that reflect asymmetry between the upper and lower halves of the cornea, IVA was significantly greater in Group 1 preoperatively, indicating an eccentric cone location. The postoperative improvement in IVA and index of height asymmetry (IHA) was greater in eyes with a single inferior ICRS (Group 1), which subsequently led to a similar refractive and visual gain as in Group 2. It has also been demonstrated that eyes with improved CDVA after ICRS implantation showed a significantly greater change in the anterior corneal surface, with relatively minor changes on the posterior surface compared with anterior corneal flattening [26]. Similarly in this cohort, Q_ant_ improved more than Q_post_, and approached to physiologic asphericity values in both groups.

Apart from cone location, stage of the disease and associated corneal curvature also have prognostic values. It is less likely to achieve significant visual gain in advanced keratoconus which has lower postoperative predictability [5]. We have also previously reported that the expected visual improvement decreases significantly when preoperative SimK_avg_ is > 55 D [9]. On the other hand, success of ICRS implantation seem to be closely related to the degree of preoperative visual limitation. Previous literature has shown that patients with good visual function at the time of surgery were more likely to lose lines of vision after the procedure, whereas patients who already had severe visual impairment were the ones that benefited the most from ICRS implantation [15]. A preoperative CDVA of < 0.5 Snellen lines was reported as a prognostic factor for gain of at least 2 lines of CDVA [27]. Likewise, we recommended ICRS surgery only for eyes with a CDVA of ≤ 0.3. As ICRS implantation might be associated with unpredictable results, just as the biomechanical behavior of a keratoconic cornea, we prefer ICRS surgery only as a last resort of visual rehabilitation in eyes with low CDVA and/ or contact lens intolerance, as an alternative to corneal transplantation which has its own inherent risks. Main advantages of ICRS implantation include reversibility and no central corneal intervention, creating no disadvantages for further possible surgical procedures [28, 29].

Limitations of this study include small sample size, retrospective evaluation of results without sample size calculation and lack of assessment of subjective satisfaction via a questionnaire after surgery. A sample size calculation was not performed as this study is a retrospective observational analysis of the authors’ patient population. Furthermore, previous studies on ICRS surgery included a similar number of eyes [13, 16, 19, 21, 22, 25]; minimum number of cases to determine a significant effect of the surgery in terms of visual, refractive or topographic parameters are not yet determined. The results may be interpreted as an early comparative analysis of a clinical observation. Future studies with prospective design and greater sample size would be necessary to validate these findings.

## Conclusions

Results of this study have shown that ICRS selection must be made custom for each cone, taking its location into consideration, to approach a regular cornea and subsequent visual improvement, postoperatively. Single ring implantation may yield satisfactory visual, refractive and tomographic results in eyes with totally asymmetric cones, regardless of the preoperative refractive error, by inducing a central shift of the cone. Double ring implantation seems to be superior in terms of improvement in keratometry, corneal astigmatism and anterior corneal asphericity values for central and partially asymmetric cones.

## Data Availability

The datasets during and/ or analyzed during the current study is available from the corresponding author upon reasonable request.

## References

[1] Kaya V, Utine CA, Karakus SH, Kavadarli I, Yilmaz AF (2011). Refractive and visual outcomes after Intacs vs Ferrara intrastromal corneal ring segment implantation for keratoconus: a comparative study. J Refract Surg.

[2] Cakir H, Utine CA. Combined Kerarings and Artisan/Artiflex IOLs in keratectasia. J Refract Surg. 2010;27:1–8. 10.3928/1081597X-20100401-02 20438024

[3] Colin J, Cochener B, Savary G, Malet F (2000). Correcting keratoconus with intracorneal rings. J Cataract Refract Surg.

[4] Utine CA, Ayhan Z, Durmaz EC (2018). Effect of intracorneal ring segment implantation on corneal asphericity. Int J Ophthalmol.

[5] Sakellaris D, Balidis M, Gorou O, Szentmary N, Alexoudis A, Grieshaber MC, Sagri D, Scholl H, Gatzioufas Z (2019). Intracorneal ring segment implantation in the management of keratoconus: an evidence-based approach. Ophthalmol Ther.

[6] Piňero DP, Alio JL. Intracorneal ring segments in ectatic corneal disease - a review. Clin Exp Ophthalmol. 2010;38(2):154–67. 10.1111/j.1442-9071.2010.02197.x 20398105

[7] Flecha-Lescún J, Calvo B, Zurita J, Ariza-Gracia MÁ (2018). Template-based methodology for the simulation of intracorneal segment ring implantation in human corneas. Biomech Model Mechanobiol.

[8] KeraRing Calculation Guidelines 2009 Version 5.2, Mediphacos Ophthalmic Professionals Inc.

[9] Utine CA, Durmaz Engin C, Ayhan Z (2018). Effects of preoperative topometric indices on visual gain after intracorneal ring segment implantation for keratoconus. Eye Contact Lens.

[10] Alpins N (2001). Astigmatism analysis by the Alpins method. J Cataract Refract Surg.

[11] Egrilmez S, Dalkılıc G, Yagcı A (2003). Astigmatizma analizinde vektoryel analiz programı [a vectorial analysis software for the analysis of astigmatism]. T Oft Gaz.

[12] Rocha G, Silva LNP, Chaves LFOB, Bertino P, Torquetti L, de Sousa LB (2019). Intracorneal ring segments implantation outcomes using two different manufacturers' nomograms for keratoconus surgery. J Refract Surg.

[13] Shabayek MH, Alió JL (2007). Intrastromal corneal ring segment implantation by femtosecond laser for keratoconus correction. Ophthalmology..

[14] Hellstedt T, Mäkelä J, Uusitalo R, Emre S, Uusitalo R (2005). Treating keratoconus with Intacs corneal ring segments. J Refract Surg.

[15] Vega-Estrada A, Alió JL (2016). The use of intracorneal ring segments in keratoconus. Eye Vis (Lond).

[16] Alió JL, Shabayek MH, Artola A (2006). Intracorneal ring segments for keratoconus correction: long-term follow-up. J Cataract Refract Surg.

[17] Chan CC, Sharma M, Wachler BS (2007). Effect of inferior segment Intacs with and without C3-R on keratoconus. J Cataract Refract Surg.

[18] Alfonso JF, Lisa C, Merayo-Lloves J, Fernández-Vega Cueto L, Montés-Micó R (2012). Intrastromal corneal ring segment implantation in paracentral keratoconus with coincident topographic and coma axis. J Cataract Refract Surg.

[19] Alió JL, Artola A, Hassanein A, Haroun H, Galal A (2005). One or 2 Intacs segments for the correction of keratoconus. J Cataract Refract Surg.

[20] Shetty R, D'Souza S, Ramachandran S, Kurian M, Nuijts RM. Decision making nomogram for intrastromal corneal ring segments in keratoconus. Indian J Ophthalmol. 2014;62(1):23–8. 10.4103/0301-4738.126170 PMC3955066 24492498

[21] Sharma M, Boxer Wachler BS (2006). Comparison of single-segment and double-segment Intacs for keratoconus and post-LASIK ectasia. Am J Ophthalmol.

[22] Miranda D, Sartori M, Francesconi C, Allemann N, Ferrara P, Campos M (2003). Ferrara intrastromal corneal ring segments for severe keratoconus. J Refract Surg.

[23] Kwitko S, Severo NS (2004). Ferrara intracorneal ring segments for keratoconus. J Cataract Refract Surg.

[24] Jarade E, Slim E, Cherfan C, El Rami H, Hassan T, Chelala E (2017). Mathematical analysis of corneal remodelling after intracorneal ring surgery in keratoconus. Int J Ophthalmol..

[25] Gatzioufas Z, Panos GD, Elalfy M, Khine A, Hamada S, Lake D, Kozeis N, Balidis M (2018). Effect of conus eccentricity on visual outcomes after intracorneal ring segments implantation in keratoconus. J Refract Surg.

[26] Sedaghat MR, Momeni-Moghaddam H, Belin MW, Zarei-Ghanavati S, Akbarzadeh R, Sabzi F, Yekta AA, Sadeghi Allahabadi J (2018). Changes in the ABCD keratoconus grade after intracorneal ring segment implantation. Cornea..

[27] Guyot C, Libeau L, Vabres B, Weber M, Lebranchu P, Orignac I (2019). Refractive outcome and prognostic factors for success of intracorneal ring segment implantation in keratoconus: a retrospective study of 75 eyes. J Fr Ophtalmol.

[28] Alió JL, Artola A, Ruiz-Moreno JM, Hassanein A, Galal A, Awadalla MA (2004). Changes in keratoconic corneas after intracorneal ring segment explantation and reimplantation. Ophthalmology..

[29] Yeung SN, Lichtinger A, Ku JY, Kim P, Low SA, Rootman DS (2013). Intracorneal ring segment explantation after intracorneal ring segment implantation combined with same-day corneal collagen crosslinking in keratoconus. Cornea.

